# Gradual and Discrete Ontogenetic Shifts in Rattlesnake Venom Composition and Assessment of Hormonal and Ecological Correlates

**DOI:** 10.3390/toxins12100659

**Published:** 2020-10-16

**Authors:** Richard B. Schonour, Emma M. Huff, Matthew L. Holding, Natalie M. Claunch, Schyler A. Ellsworth, Michael P. Hogan, Kenneth Wray, James McGivern, Mark J. Margres, Timothy J. Colston, Darin R. Rokyta

**Affiliations:** 1 Department of Biological Sciences, Florida State University, Tallahassee, FL 32304, USA; rs17c@my.fsu.edu (R.B.S.); emh18b@my.fsu.edu (E.M.H.); sellsworth@bio.fsu.edu (S.A.E.); mhogan@bio.fsu.edu (M.P.H.); kpwcrotalus@gmail.com (K.W.); jamesjmcgivern@gmail.com (J.M.); margres@usf.edu (M.J.M.); tim@maddreptiles.com (T.J.C.); drokyta@bio.fsu.edu (D.R.R.); 2 School of Natural Resources and Environment, University of Florida, Gainesville, FL 32611, USA; nmclaunch@ufl.edu; 3 Department of Organismic and Evolutionary Biology, Harvard University, Cambridge, MA 02138, USA; 4 Department of Integrative Biology, University of South Florida, Tampa, FL 33620, USA

**Keywords:** ontogeny, size-structured population, *Crotalus horridus*, *Crotalus adamanteus*, testosterone, venom

## Abstract

Ontogenetic shifts in venom occur in many snakes but establishing their nature as gradual or discrete processes required additional study. We profiled shifts in venom expression from the neonate to adult sizes of two rattlesnake species, the eastern diamondback and the timber rattlesnake. We used serial sampling and venom chromatographic profiling to test if ontogenetic change occurs gradually or discretely. We found evidence for gradual shifts in overall venom composition in six of eight snakes, which sometimes spanned more than two years. Most chromatographic peaks shift gradually, but one quarter shift in a discrete fashion. Analysis of published diet data showed gradual shifts in overall diet composition across the range of body sizes attained by our eight study animals, while the shifts in abundance of different prey classes varied in form from gradual to discrete. Testosterone concentrations were correlated with the change in venom protein composition, but the relationship is not strong enough to suggest causation. Venom research employing simple juvenile versus adult size thresholds may be failing to account for continuous variation in venom composition lifespan. Our results imply that venom shifts represent adaptive matches to dietary shifts and highlight venom for studies of alternative gene regulatory mechanisms.

## 1. Introduction

Ontogenetic shifts are common in nature [1], and can optimize resource use, defense, or other ecological function across life stages when they are adaptive. Optimal trait performance is expected to differ most across life stages in organisms that metamorphose, or in size-structured populations that are defined by altered patterns of resource use throughout development [2]. Ontogenetic shifts can be described by the nature of their trajectory: namely, whether they occur suddenly or gradually [3]. Discrete ontogenetic shifts involve a number of rapidly occurring alterations, such as in the metamorphosis of insects [4] or the shift in the diet of juvenile yellowfin tuna when they reach 1.5 kg [5], and correspond to sudden changes in life-history features [6]. Gradual shifts are known from many plant species, where defense traits such as palatability to herbivores and phytochemical profiles change slowly [7]. Gradual ontogenetic shifts in animal phenotypes that match gradually shifting resource use would provide models for understanding the proximate and ultimate causes of gradual ontogeny in animals’ systems. 

Ontogenetic shifts are well-known in the venom composition of animals such as snakes [6,8,9,10,11,12,13,14,15,16,17,18,19] and scorpions [20]. Animal venoms are composed of an assortment of proteins and peptides that are produced in a gland and injected into prey or in defense against predators [21]. Ontogenetic shifts in composition can yield measurable variation in venom function as stark as shifts from primarily neurotoxic and myotoxic to primarily hemotoxic and hemorrhagic venoms in snakes [22,23,24]. Venom ontogeny has medical relevance, as snakebite outcomes and antivenom efficacy can depend upon venom composition [25,26,27,28]. It also has biological relevance, as ontogenetic shifts in venom composition can impact the ability of snakes to incapacitate certain species of prey [22]. Although the presence of shifts has been demonstrated across many species, less is known about their tempo, either as gradual or sudden processes, and the proximate physiological signals that might initiate shifts in venom composition remain largely unexplored [10,29]. 

Studies on both the evolution and biomedical application of venom often categorize animals as juvenile or adult based on size cutoffs, such as size at reproductive maturity [24,30,31,32,33,34,35]. This logic inherently implies a rapidly shifting mode for the ontogenetic shift in venom composition. If the trajectory of the shift were more continuous, this would confound studies of venom variation by introducing variation from mid-sized animals that blurs the lines between the juvenile and adult study groups. Previous work in Pacific rattlesnakes (*Crotalus oreganus ssp.*) suggested that protease activity in venoms begins to increase between 50 and 60 cm in snout–vent length (SVL) [22], while in *Crotalus adamanteus* there is evidence for a shift in venom composition at a length of 102 cm, the size that indicates sexual maturity in the species [30]. Prior works on ontogenetic shifts in venom across developmental stages tend to employ a “venom census”, surveying populations by collecting single samples from many individuals of different sizes. However, the census approach is limited by both significant individual variation in venoms [32,36], the potential presence of a terminal venom phenotype in otherwise indeterminately growing snakes [37], and the potential for size-biased sampling. Therefore, census or population-snapshot surveys cannot account for potential individual variation in tempo and mode of ontogenetic shifts, obscuring our ability to distinguish between gradual and discrete shifts in venom phenotype. The problem of individual variation in the ontogenetic trajectory and overall venom phenotype is compounded if there is indeed a terminal phenotype, beyond which further growth is no longer accompanied by shifts in venom. In a venom census, terminal phenotypes appear to represent sudden change followed by a cease in change, but whether or not apparent changes are gradual from neonate to subadult and adult sizes is difficult to determine. Time-series data on captive individuals that span small juvenile to large adult sizes would control for extraneous factors and individual variation to allow a detailed look at the trajectory of venom compositional change. 

The timber rattlesnake (*Crotalus horridus*) and eastern diamondback rattlesnake (*Crotalus adamanteus*) are heavy-bodied pit vipers native to the southeastern United States that reach adult body sizes over 1 m in length. Both species undergo ontogenetic shifts in venom composition [16,18,30]. We profiled the shift in venom composition across the lifespan of three individual eastern diamondback rattlesnakes (*C. adamanteus*) and five timber rattlesnakes (*Crotalus horridus*) including representatives of Type A (neurotoxic) and Type B (hemorrhagic and hemotoxic) phenotypes [38]. By assessing changes in proportion of venom proteins and shifts in chromatographic peak abundances within individuals over time, we characterize the mode and tempo of ontogenetic changes in venom of two rattlesnake species. Additionally, we investigated correlations between changes in venom composition and serum testosterone to explore the hormone’s potential as a mediator of ontogenetic venom shifts, as testosterone expression also exhibits ontogenetic changes with body size in pit vipers [39,40]. Under the hypothesis that the shift in venomic composition happens discreetly at a distinct size [30], we would predict that the majority of the magnitude of compositional change should occur over a brief window of time. In contrast, the gradual change hypothesis predicts slower continuous change in composition as snakes grow from small juveniles to large adults, possibly with a plateau when a terminal adult phenotype is reached. Finally, when venom ontogeny is adaptive, we would expect shifts in snake diet composition that are similar in mode to those of venom. We examined this possibility by mining diet data for both species from previously published studies of gut contents of wild specimens and determine the mode of ontogenetic shifts in diet as snakes grow.

## 2. Results & Discussion

### 2.1. Venom Composition Changes Slowly over Time

**Figure 1:**
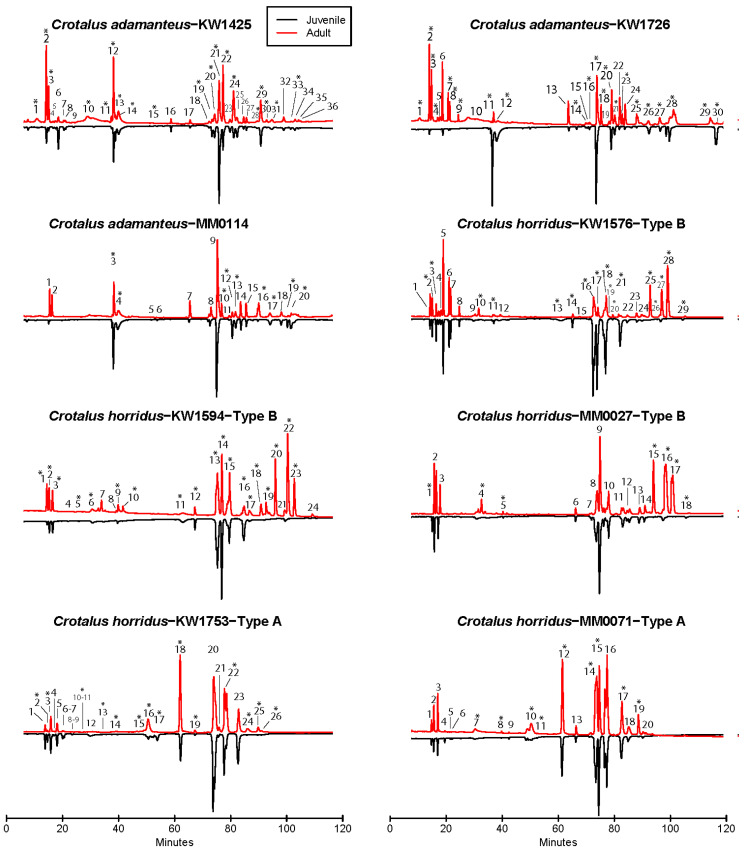
High performance liquid chromatography (HPLC) profiles of *C. adamanteus* (*n* = 3) and *C. horridus* (*n* = 5) venom samples at 220 nm. The black line reflected into the negative absorbance range shows the profile for the first sample taken as a juvenile, while the red line shows the last sample taken as a large adult. An asterisk indicates significant ontogenetic variation (*p* < 0.05). Peak height corresponds to absorbance at 220 nm, but heights were scaled to max peak height = 1 and absorbance not shown for simplicity.

**Figure 2:**
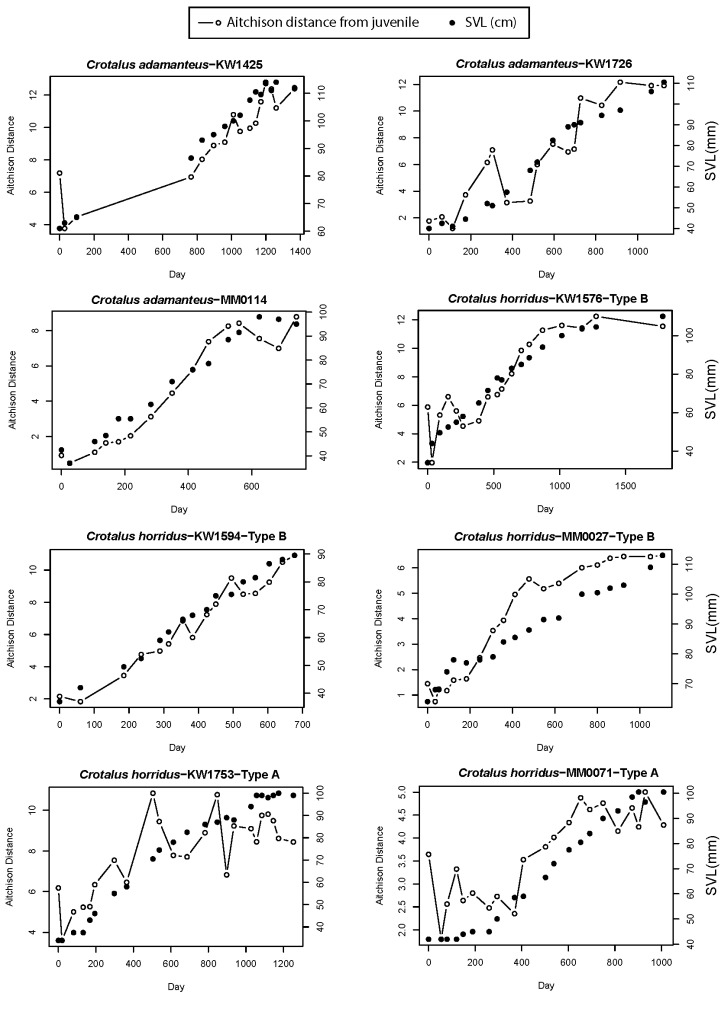
Mode and tempo of venom ontogeny. Line graphs show the change in both venom composition (as Aitchison distance from initial samples) and snout-to-vent length over time for all eight snakes studied. Open circles show venom Aitchison distance from early juvenile average, while filled circles show contemporaneous body size (SVL).

We detected statistically significant ontogenetic shifts in overall venom composition in all individual snakes in this study using permutational mutivariate analysis of variance (PERMANOVA) (Figure 1, Table 1). Time explained between 34 and 80% of multivariate venom variation in these snakes. Time series plots of Aitchison’s compositional distance from the small juvenile venom phenotype (Figure 2) to that of latter samples revealed ontogenetic change that occurs incrementally over years as snakes grew in body size, where shifts sometimes reached a plateau as snakes became very large adults (e.g., Figure 2, snake KW1576). We classified the mode of ontogenetic shifts as linear, sigmoid-gradual (occurs over more than the time of a single active season based on slope coefficient), and sigmoid-discrete (occurs in less than the time of a single active season), we found that the linear model was the top model in four of the eight snakes examined, although the linear model was significantly better than the sigmoid model (DeltaAIC > 2) in only a single case (Type B *C. horridus* KW1594; Table 2). The sigmoid model was significantly better than the linear model in the other four individuals (Table 2). Of these four cases, only one was classified as showing a rapid, discrete shift (Type B *C. horridus* MM0027; 242 days); the other three cases showed evidence of a gradual shift that would take longer than a single active season, although KW1753 (Type A *C. horridus*) was more rapid than others (309 days; Table 2). Similarly, the linear model was the best fit (but not significantly better than sigmoid, DeltaAIC = 0.6) for MM0114 (*C. adamanteus*), but this individual also showed a rapid change (213 days; Table 2). The tempo for the other five individuals was substantially longer (≥677 days; Table 2). Overall, we found evidence for gradual shifts in six of the eight snakes tested (four of which were more than one year in length) with MM0114 and MM0027 (and, to a lesser extent, KW1753) showing evidence of discrete ontogenetic shifts. Some unique characteristics of these two snakes are worth noting. MM0114 is unique among the three sampled *C. adamanteus* individuals studied herein, in that venom sampling ended before the snake reached 100 cm SVL. Margres et al. [30] estimated that juvenile and adult venoms can be differentiated in wild snakes best at 100 cm SVL, and so a significant portion of this animal’s ontogenetic change may have been yet to come at the conclusion of sampling. Finally, MM0014 is from a different population than the other *C. adamanteus*, so shifts could occur differently in different populations. Snake MM0027 differs from the seven other snakes in this study in that it was the largest individual at time of first sampling; it was wild caught and first sampled at 64 cm in length. Based on the profiles of the two other Type B *C. horridus* (which were first sampled when only half the size of MM0027 at capture), we likely missed between ⅓ and ½ of the ontogenetic shift experienced by MM0027, suggesting that earlier sampling may have led to detecting a gradual shift in this animal.

**Figure 3:**
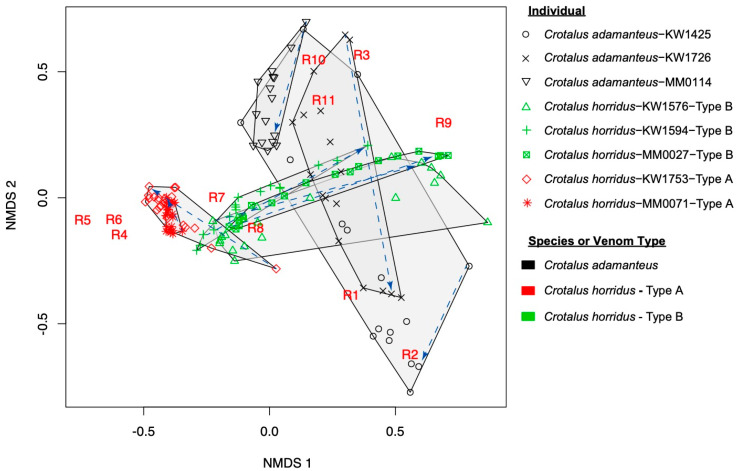
Non-metric multidimensional scaling of venom phenotypes binned into eleven retention time blocks (R1–R11) to allow comparison across all individuals and samples. Polygons are minimum convex shapes enclosing all samples from a single individual snake. Blue dashed arrows point from the earliest sample taken to the last for each individual. HPLC retention time window centroids are also plotted on the chart to clarify the relative position of the samples in venom space. The retention time windows used are as follows for each region designation: R1 (10–20 min), R2 (20–30 min), R3 (30–40 min), R4 (40–50 min), R5 (50–60 min), R6 (60–70 min), R7 (70–80 min), R98 (80–90 min), R9 (90–100 min), R10 (100–110 min), and R11 (110–125 min).

We used non-metric multidimensional scaling of binned high performance liquid chromatography (HPLC) retention time blocks to allow between-individual comparison and visualize the position of all samples from all snakes in a single multivariate space (Figure 3). Such comparisons were not possible before binning as homologous peaks were called within, but not between, individuals. Although sample size limited our ability to statistically compare movement among individuals through venom space, several patterns are apparent. First, *C. adamanteus* and both venom types of *C. horridus* occupied unique areas of venom phenotype space, and the major directions of within-individual variation in each species (or venom type) tend to have a similar pattern of orientation. For example, within-individual variation in the three *C. adamanteus* is oriented largely along NMDS axis 2, whereas type B *C. horridus* venom varies along NMDS axis 1. Meanwhile, the two type A *C. horridus* vary over an apparently smaller overall space, and that variation is oriented diagonally along Figure 3, and therefore lies along both axes. In seven of eight individuals, we again see apparent effects of ontogeny driving within-individual variation, as the first and last samples taken in time tend to polarize each individual in the NMDS space (beginning and end of blue arrows in Figure 3). The exception is the KW1425, which was wild-caught at moderate size (61 cm SVL). The first sample taken from this snake occupies a unique area of venom space, whereas the second and third samples show the same trend as seen in the other seven individual snakes, in that they are the two samples with the highest NMDS axis 2 values, and so lie furthest away from the samples taken as a large adult. The first sample taken from KW1425 could be therefore be biased by uncontrolled impacts of initial capture and prior free-living.

The clear implication of these analyses is that ontogenetic shifts in the complete venom profile are not wholly discrete processes. Instead, the change in composition from a neonate to adult venom tends to occur across a longer period of time. It is important first to acknowledge our small overall sample size of eight individual snakes (two species) as a limitation in our ability to generalize our findings as representative of each species, and particularly to rattlesnakes as a group. However, we are confident in the power of our repeated samples to provide useful information about the individual with high resolution, and similar gradual shifts across those individuals over which we had the most experimental control encourages the view that gradual ontogenetic shifts are commonplace and worth further exploration. The passing of time, which is likely a proxy for increases in body size, led to increasing Aitchison distance from the neonate baseline phenotype with 90% of observed change being completed in 213–989 days (Table 2). Post-hoc model comparison of time vs. body size as predictors of venom compositional change revealed that body size was a better predictor of venom change than time in five of the eight snakes studied here; body size and time explained a comparable amount of overall variation in the models (Supplemental Table S1). The result that body size can be a better predictor than time suggests a direct role of growth in venom compositional shifts despite the fact that body size measurements were subject to clear observer error associated with measuring an unanesthetized restrained snake (visible in how some SVLs appeared to decrease between subsequent measurements in Figure 2). Most samples appear to reach a plateau in amount of differentiation from the neonate venom phenotype, indicating that a terminal adult phenotype does exist; the path toward that phenotype is generally (but not always) achieved through gradual shifts in protein composition that eventually reach a plateau where change drastically slows or ceases. The power of body size as a covariate for venom compositional change is encouraging for researchers studying free-ranging snake populations, as age cannot be determined directly but body size can be easily measured.

The slow tempo of compositional change over time and body size in at least half of our samples challenges the notion that ontogenetic shifts occur discretely in *C. adamanteus*, as proposed by Margres et al. [30]. There are also multiple plausible biological explanations for the distinction between this study and that of Margres et al. [30]. First, most studies focused on the evolution of venom categorized individuals as being juvenile or adult based on predetermined size cutoffs in terms of total SVL [31,34]. The issue with this practice is that it inherently implies that a sudden ontogenetic shift in composition occurs once the size cutoff between designations is reached, and thus omits the potential gains of treating body size as a continuous predictor of venom variation. In our study, we leverage continuous measurement of size and sampling of venom variability. Second, any individual or geographic variation in the timing or magnitude of the ontogenetic shift would further hinder attempts to discern the tempo of shifts in census-type studies. Our study collected time-series based data over the course of several hundreds of days from individuals in captivity and controlled for unknown factors such as geography, diet, and sex by conducting analyses within single individuals. Maintaining specimens in this manner permitted concrete observation of the tempo and mode at which changes were occurring. Margres et al. [30] used a fairly restrictive definition to delineate support for the hypotheses of gradual vs. discrete shifts in multivariate venom composition, namely whether or not a sigmoid fit outperforms a linear fit of body size to venom composition across the full range of body sizes sampled from free-ranging snakes. We show here that sigmoid best fits are expected to explain overall venom composition, particularly if a terminal phenotype is eventually reached but the snake continues to grow. These sigmoid patterns of venom variability across lifespan can still take quite long to complete, which would not be obvious from sampling only wild-caught individuals a single time.

### 2.2. Peak-by-Peak Analysis Reveals Both Modes of Expression Variation

**Figure 4:**
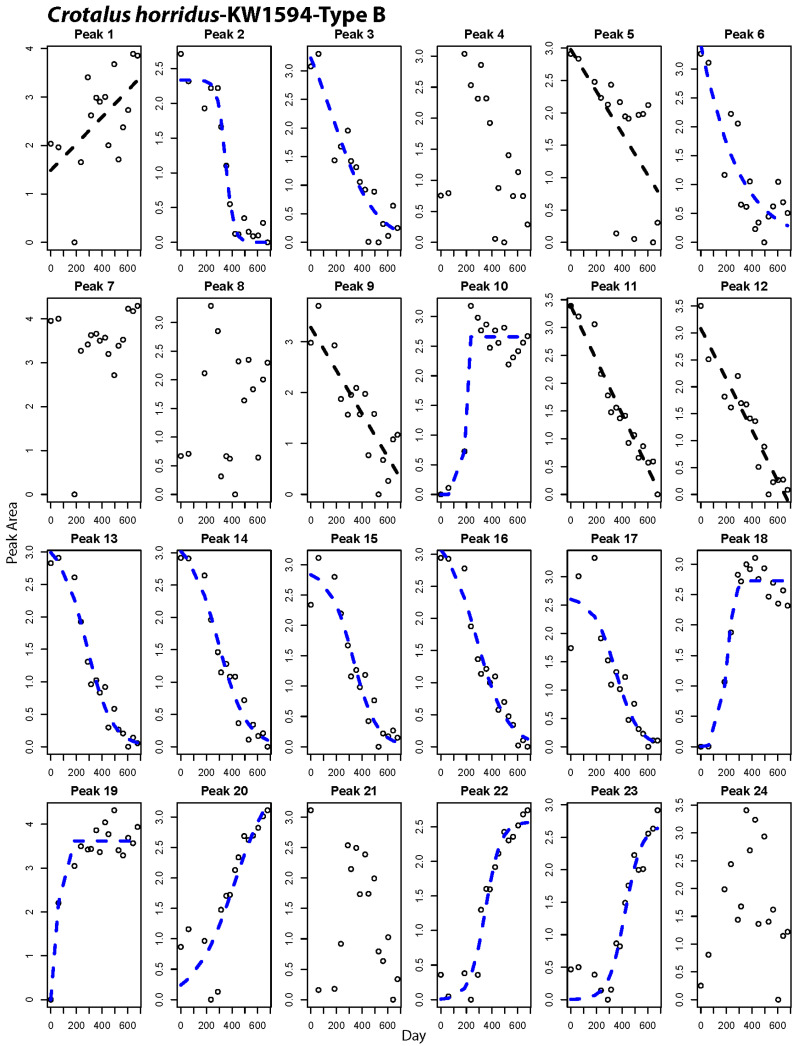
Peak-by-peak analysis of relative abundance shifts over time for a Type B *Crotalus horridus*. Panels with best-fit lines indicate peaks that changed significantly in abundance over time. Black lines indicate best fit of a linear model to the data, while blue lines show cases where a non-linear fit was best.

**Figure 5:**
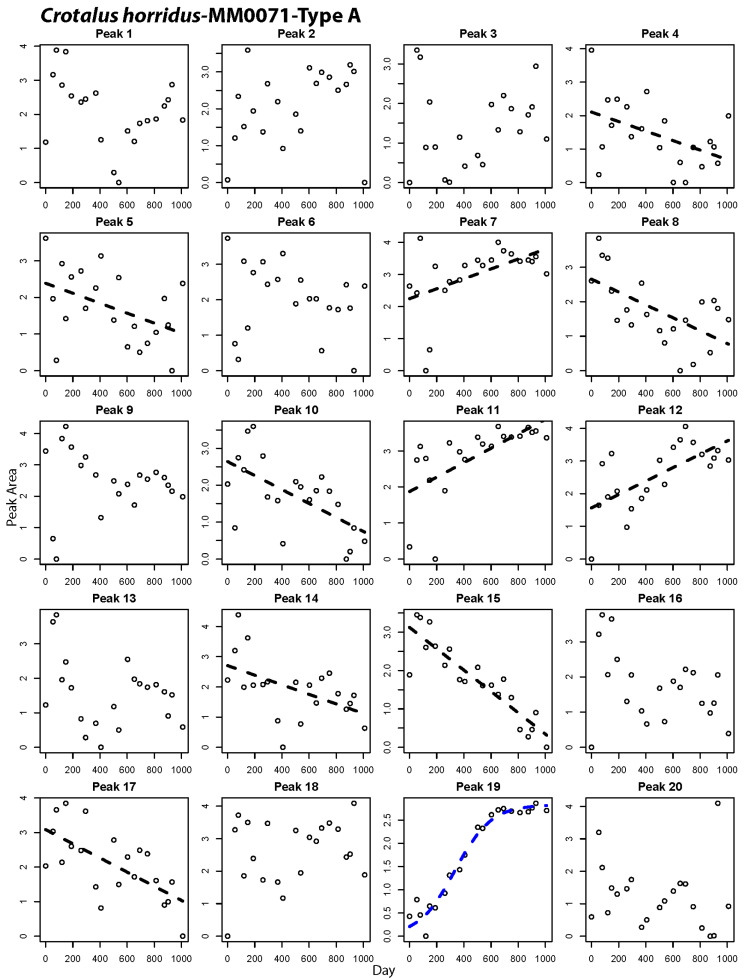
Peak-by-peak analysis of relative abundance shifts over time for a Type A *Crotalus horridus*. Panels with best fit lines indicate peaks that changed significantly in abundance over time. Black lines indicate best fit of a linear model to the data, while blue lines show cases where a non-linear fit was best.

**Figure 6:**
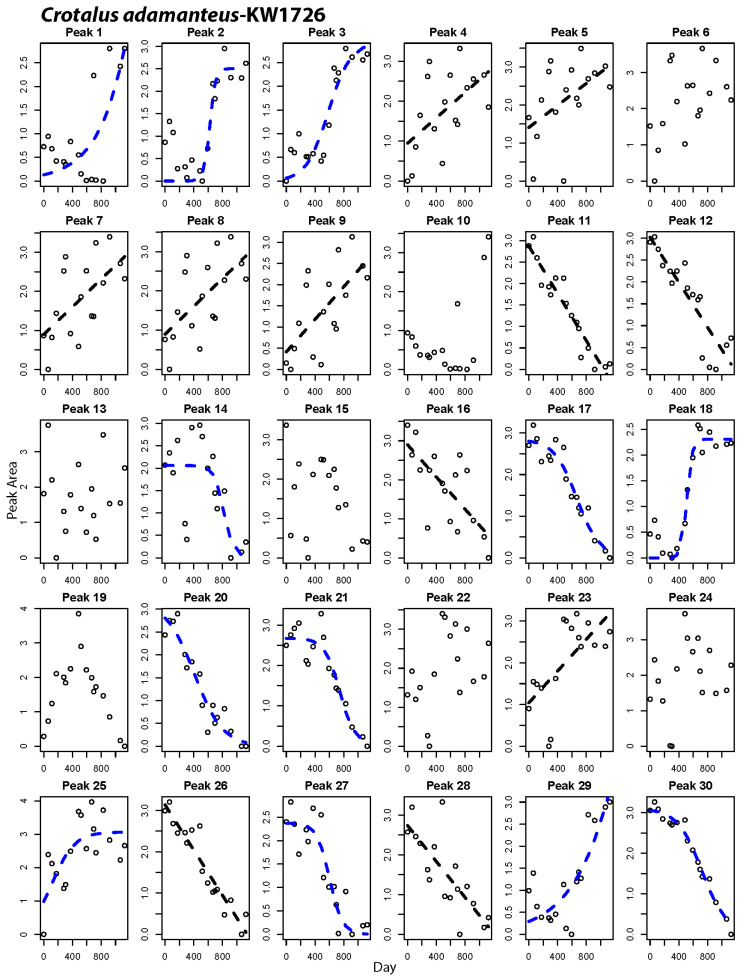
Peak-by-peak analysis of relative abundance shifts over time for an individual *Crotalus adamanteus*. Panels with best fit lines indicate peaks that changed significantly in abundance over time. Black lines indicate best fit of a linear model to the data, while blue lines show cases where a non-linear fit was best.

Gradual shifts in overall venom composition could result from many gradually shifting peaks, or multiple discretely shifting individual peaks whose shifts occur at different time points. Our repeated sampling within the same individuals permitted peak-by-peak analysis of compositional shifts, allowing an estimate of the overall percentage of venom proteins that shift expression levels and determine if both gradual and discrete modes of expression variation operate among venom genes. These analyses showed that ontogenetic shifts involved large numbers of venom proteins, with between 55% and 89% of the peaks changing significantly in relative abundance over time (Table 3, peaks marked with asterisks in Figure 4). We found evidence for both gradual and discrete modes of shift among the peaks within individual snakes (Table 3; Figure 4, Figure 5 and Figure 6, Supplemental Figures S9–13). On average, 25% of peaks showed discrete shifts. A discrete shift is exemplified by Peak 18 in Figure 6, which shifted from a low abundance juvenile state to a maintained high abundance in adulthood over the course of three sampling periods spanning 221 days. Some of the gradual shifts could be artifacts of our use of chromatographic peaks as measures of abundance since a single peak may contain multiple proteins. It is therefore possible that multiple discrete shifts spaced over time could generate an overall pattern of gradual change in peak relative abundance. We consider this unlikely to explain the bulk of our observations, given the fact that the observed transitions in relative abundance of many peaks are markedly smooth in the nature of shifts (e.g., Figure 4, peak 18, Figure 5, peak 19) and a given peak usually contains no more than 3 protein isoforms [10]. Future work that combines transcriptomic and mass-spectrometric characterization of these changes could rule out varied peak contents as an artifact and reveal whether the mode and tempo of shifts varies among venom protein families.

The repeated-samples nature of our study allowed for the control necessary to call peaks in a highly standardized fashion for each individual, providing the resolution to visualize slow shifts in peak abundance over time and classify their mode of shift based on our statistical framework. Previously, proteomic analysis of *C. adamanteus* and *C. horridus* described by Wray et al. [10] identified HPLC peak contents based on shared peptide evidence. Their analyses included individuals or siblings included in the present study, and therefore permit us to infer venom protein contents of peaks in this study (Supplemental Tables S2 and S3). The protein contents of these peaks indicate that gradual shifts occur for peaks containing multiple important venom gene families, including snake venom metalloproteinases (SVMP), snake venom serine proteases (SVSP), phospholipases A2 (PLA2), bradykinin potentiating peptides (BPP), myotoxins (MYO), and C-type lectins (CTL). While identification of specific protein contents of peaks was not a goal of our study, we emphasize the usefulness of combining serial sampling as done here with mass-spectrometric approaches for a more detailed understanding of whether different modes of shift tend to occur in different gene families. Such a pattern would suggest alternative regulatory mechanisms among gene families.

Margres et al. [41] discussed the compositional (simplex) nature of the venom phenotype extensively, pointing out that a rise in relative abundance of one protein in the venom necessitates a drop in abundance for other proteins. Within the framework of compositional data, it is important to remember that the shifts in relative abundance we see do not necessitate variation in transcription or translation of a protein across an animal’s life span. Instead, major up- or down-regulation of other venom genes can change the relative abundance of other proteins in a compositional phenotype such as venom. In other words, a relationship between time and peak relative abundance like that for KW1594 peak 11 (Figure 4; containing VEGF and NGF) can be the result of two distinct processes. First, the action of cis- or trans-regulatory mechanisms may be slowly reducing transcription over time in one or both of these genes, as post-transcriptional regulation of expression is not a large contributor to venom variation [42], leading to decreased production of this peak relative to other peaks that are produced at a constant rate. Alternatively, the reverse can be true, where upregulation of other genes via gene regulatory mechanisms is leading to a slow depression in relative abundance of these proteins in the venom, despite constant transcription and translation. It may be that those peaks displaying the steepest slopes, such as KW1594 peak 22 (Figure 4) are the most likely candidates to be targets of regulatory mechanisms over the course of development. Studies combining transcriptomics and proteomics with novel tools to study gene regulation are forthcoming in snake venomics [43,44] and will shed light on the proportion of venom that is directly regulated vs. impacted in a relative manner by changes in other venom components. More broadly, the presence of ontogenetic shifts in peak abundance that vary from highly linear to highly discrete and sigmoidal in shape suggests that alternate regulatory mechanisms are at play among the genes contributing to the same predatory phenotype of venom. Our work here therefore indicates that venom provides ostensible targets for studies of different gene regulatory mechanisms operating within a common functional module.

### 2.3. Assessing Testosterone as a Predictor of Venom Change

**Figure 7:**
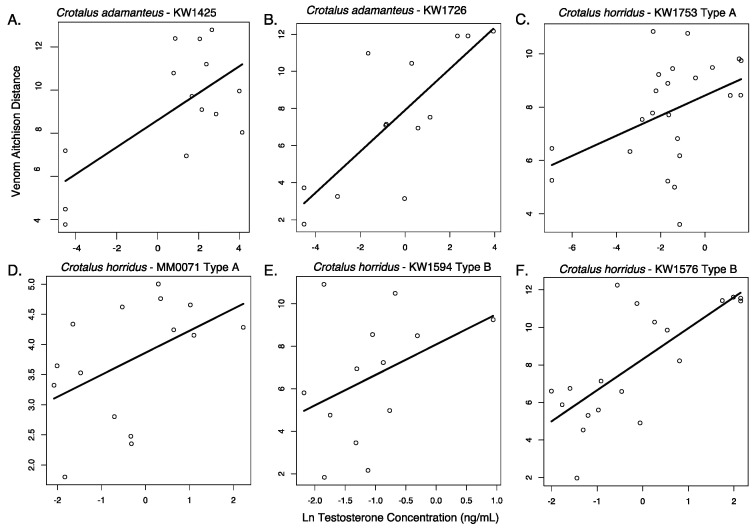
Relationship between venom compositional change (Aitchison distance from juvenile phenotype) and natural log-transformed testosterone concentration for six rattlesnakes raised in controlled laboratory conditions, including two *Crotalus adamanteus* (**A**,**B**), two Type A *Crotalus horridus* (**C**,**D**), and two Type B *Crotalus horridus* (**E**,**F**).

Our results do not meet the prediction of a strong linear relationship between the change in venom composition and the testosterone concentration across the course of our study (Figure 7), and a high correlation between body size and testosterone violates the assumption of a lack of multicollinearity in predictors, preventing us from conducting informative multiple regression analysis with both body size and testosterone level in the models. The change in venom composition and ln-testosterone concentration were significantly correlated in four of the six snakes for which testosterone was assayed (KW1425: *R*^2^ = 43%, *p* = 0.01; KW1576: *R*^2^ = 55%, *p* = 0.0003; KW1753: *R*^2^ = 18%, *p* = 0.04; KW1726: *R*^2^ = 62%, *p* = 0.001; MM0071: *R*^2^ = 23%, *p* = 0.07; KW1594: *R*^2^ = 15%, *p* = 0.18). These significant correlations underscore the patterns of maturation in both these snake species, where both testosterone concentrations and venom distance from the juvenile venom phenotype increase as snakes mature. We expected both venom to change and testosterone to increase into adulthood, as ontogenetic changes in venom composition are known from both species studied [18], and testosterone is known to increase with body size in snakes [39,40,45] as it mediates sexual maturity in rattlesnakes in both sexes [46]. As a result, a correlative study like ours required a specific prediction of a very strong relationship. Anything but a very strong linear relationship can be explained by other factors. Clearly, Figure 7 shows that the samples taken during periods of high testosterone are also samples that have changed in venom composition from the earliest sampling periods. However, the range in Aitchison distances associated with moderate to high testosterone levels spans nearly the full spectrum of the ontogenetic shift in venom. While seasonal testosterone shifts are known from adult rattlesnakes [46,47,48,49,50], there is little information on seasonal testosterone in young, small individuals [39]. Therefore, we suspect that the patterns in Figure 7 are best explained by slow ontogenetic shifts in venom that clearly occur over the snakes’ lifespans (Figure 2) and that these are eventually accompanied by changes in testosterone as snakes reach a size of sexual maturity.

The proximate physiological causes of intra-individual venom variation have received very little attention, despite the potential of such information as a tool in both studies of venom gene regulation and in proper care of animals used in venom production for medical uses. Our investigation of testosterone as a possible physiological trigger of venom variation is among the first to assess the proximate factors inducing the extreme changes seen in animal venoms [12,51]. Generally, our lack of power to draw stronger conclusions about the potential for a relationship between venom and testosterone reflects how our study, and any correlative study that involves hormones with complex roles, will be limited compared to an experimental manipulation of hormone levels. We emphasize that our inconclusive testosterone results are thus a demonstration of how experimental manipulations, instead of purely correlative studies, are the key to uncovering the proximate causes of ontogenetic shifts in venom. Experimental manipulation of levels of several metabolic and maturation hormones is now commonplace in reptiles [29,39]. Future studies that manipulate hormone levels in neonate snakes and conduct before and after sampling of venom will be particularly valuable in uncovering the triggers of shifts in venom phenotypes as snakes grow.

### 2.4. Mode of Dietary Shifts

**Figure 8:**
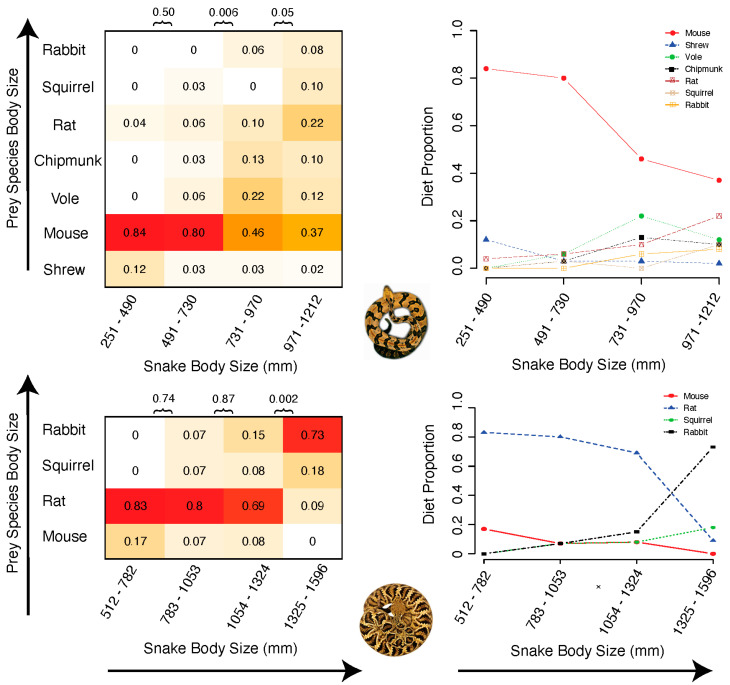
Size-structured dietary ecology in two rattlesnakes. Both *Crotalus horridus* (top) and *Crotalus adamanteus* (bottom) show ontogenetic shifts in the contribution of different mammalian prey items to their diet. Diet items from Clark et al. (2002) and Means et al. (2017) show differences among some body size quartiles. Data in cells are proportions of total diet (darker color = greater proportion), and values above each matrix are p-values for chi-squared tests for differences in counts of prey items between adjacent size class quartiles.

We found evidence that ontogenetic changes in diet reported in prior studies occurred across body size in *C. horridus* (*n* = 179 total prey counted), where there were significant differences in diet composition between both the 2nd and 3rd body size quartiles (*p* = 0.006), as well as between the 3rd and 4th (chi-square test; *p* = 0.05, Figure 8). The shift of diet in *C. horridus* manifests as consumption of shrews and mice at small sizes, with gradual increases in the abundance of larger mammals such as squirrels and rats at larger body sizes. Inspecting the shifts in abundance of the individual prey categories suggests that alternative modes of dietary shifts may occur within *C. horridus*. Rat and vole abundances show relatively steady increases over time, while mouse abundances shift in a discrete fashion between the 2nd and 3rd body size quartiles. The overall nature of the shift in *C. adamanteus* is more discrete in nature (Figure 8), with significant compositional variation between only the 3rd and 4th body size quartiles (*p* = 0.002), although failure to detect differences among smaller size classes could be due to low power (*n* = 58 total prey counted with particularly low numbers in the smallest size class). The 4th size quartile present in the diet study actually lies outside the body size range of the snakes whose venom we sampled in this study, meriting examination of the taxon-specific patterns of abundance variation in the 1st through 3rd size quartiles. There is a propensity toward the consumption of larger prey taxa over time in *C. adamanteus*, evidenced by the *C. adamanteus* diet exclusively (100%) consisting of mice and rats at the 512–782 mm range whereas rabbits and squirrels appear in the 2nd size quartile (783–1053 mm), and rabbits double in abundance between the 2nd and 3rd quartiles.

As dietary breadth has been previously shown to impact venom composition in taxa such as *Echis* vipers and *Sistrurus* rattlesnakes [8,12], the specific nature of dietary shifts such as those in *C. adamanteus* and *C. horridus* may be expected to predict the mode and tempo of venom ontogeny, but like the mode and tempo of venom shifts, the nature of dietary variation is complex, with the overall nature of these shifts underscored by apparent gradual and discrete shifts in the abundance of individual prey types. Our ability to link dietary shifts to the mode of venom shifts is likely limited by the differential scope of the data collected, as we compare range wide diet data with venom sampled from individuals from a single region in the southeastern U.S. Given the evolutionary lability of snake venoms such as those of *C. adamanteus* [30], it may be that local diet studies may provide an even stronger match to the mode and tempo of venom ontogeny. High resolution diet studies now exist for several species of vipers [52,53,54], and these studies often include information about the body size of snakes that had consumed particular prey items. Notably, neutrality or near-neutrality has not been rejected on a protein-by-protein basis for any venom, so functional studies of venom ontogeny’s impact on prey toxicity will be required to clearly demonstrate the hypothesized adaptive nature of venom ontogeny as a phenotypic match to a changing diet. Future studies that combine diet data with profiling of the mode and tempo of ontogenetic shifts in the venom of each snake species would allow a phylogenetic comparative test of the hypothesis that the ontogenetic shifts in venom evolve to match the nature of a snake species’ movement through a dietary ecological niche. Additionally, treating the presence or strength of ontogenetic shifts as a trait for population-level comparisons may help differentiate adaptive and neutral explanations for ontogenetic venom variation among venom proteins [32].

## 3. Conclusions

Ontogenetic shifts in snake venom are more the rule than exception, changing the functional profile of venom in both biologically and biomedically important ways [22,27,55,56]. Our study is the first to undertake venom compositional profiling at the level of an individual spanning neonate to large adult sizes with a high density of repeated samples, which allowed several novel insights into the nature of the ontogenetic shifts in the species studied. In particular, we show multiple shifts in venom composition that occurred gradually over the course of over 1 m, or multiple years, of growth. The vast majority of previous work on ontogenetic shifts in venom represent population sampling efforts, where venom traits are regressed against individual body size, and therefore leave individual and population variability as an uncontrolled variable [22]. Studies such as those are used in documenting the presence and intensity of a shift, but the presence of extensive individual variability in venoms [36] leads to poor inference into the specific nature of ontogeny. Still, many studies of venom in both natural and laboratory settings merely report venoms as juvenile or adult, and thus bin highly continuous variation in venom traits over lifespan into groupings of binary predictor variables.

We therefore suggest that one of the most important implications of our work is the need to record more detailed life history data, particularly body size, as venom is collected. The only comparably long-term study to ours involving another large pit viper (*Bothrops atrox*) provides a demonstration of the utility of considering body size information when making inferences about venom ontogeny [57]. These authors captured snakes in the wild classified as adults and used HPLC and enzymatics to profile change in venom during long-term captivity. While minor compositional shifts were reported in all snakes, only 4 of 13 snakes showed shifts in venom proteins classified as “core function toxins”, and the authors provided several potential explanations for the major changes in these select individuals, including dietary shifts, collection during the rainy season, and that two of the four snakes were the two smallest females collected. Repeated measurements of body size were not provided in the study by Amazonas et al. [57], but three of the four animals showing the large compositional shifts were indeed three of the four animals with the smallest body sizes at collection. Therefore, extrapolating our results in *Crotalus* to *Bothrops* leads us to hypothesize that these animals may have simply had the most opportunity for greater compositional change in venom, whereas the larger animals were already close to their terminal adult venom phenotype at first collection.

Animal venom provides a unique window into the path from genotype to phenotype to function, as the trait is broadly accessible through modern ‘omic’ and enzymological technologies [9,58]. Venomous snakes in particular are accompanied by extensive studies of their behavioral [59], physiological [39,60,61], and dietary ecology [22,52,54]. Venom systems therefore present an incredible opportunity for integrative biology that addresses both the proximate and ultimate causes of variation in a key functional trait, as long as venom research is done in conjunction with collecting quality life-history information. Shifts in venom are likely adaptive responses to shifts in diet that are natural consequences of gape-limited predation in snakes. Closely related species of venomous animals that show extreme variation in the range of body sizes experienced and the steepness and intensity of ontogenetic shifts in diet represent fruitful ground for studying the genomic basis and evolution of ontogenetic shifts in venom, and will inform us further on the interplay between the evolution of development and the evolution of diversity.

Data accessibility: raw data are provided as supplemental information.

## 4. Materials and Methods

### 4.1. Acquisition of Specimens and Samples

Venom samples (*n* = 148 total samples) were obtained from three *C. adamanteus* and five *C. horridus*. Individuals were either originally caught in Florida or Georgia (Supplemental Figure S1) or raised from previously established cohorts at Florida State University; detailed specimen information is provided in Table 4. These snakes were maintained in captivity for multiple years, growing from juvenile size (newborn or wild caught juveniles) to large adult sizes. Collection and handling of these animals was approved under Florida Fish and Wildlife Conservation Commission permits LSSC-13-00004, LSSC-09-0399, and LSSC-12-00071A and Institutional Animal Care and Use Committee (IACUC) protocols #0924 and #1333. We collected venom and blood samples every second month by inducing snakes to bite through a parafilm-covered beaker, and venom was then lyophilized and stored at −80 °C until use. Blood samples were centrifuged at 1500× *g* for 10 min, and serum was stored at −40 °C.

### 4.2. High-Performance Liquid Chromatography

Venom samples were redissolved in LCMS water and diluted to a concentration of 0.18 mg/mL. We determined venom protein concentration by the A280 reading from a NanoDrop Photospectrometer (ThermoFisher Scientific, Waltham, MA, USA). We then loaded 15 μg of venom protein onto a Prominence HPLC apparatus (Shimadzu Corp., Columbia, MD, USA). Samples were run through a Phenomenex Aeris widepore column (3.6 micron, C18, part # 00G-4482-AN) maintained at 25 °C. Samples were eluted with solution A as 0.1 mM TFA in water and solution B as 0.06 mM TFA in acetonitrile, and absorbance at 220 nm was measured over 150 min with the following run parameters: 5 min at 10% B, 110 min increasing from 10 to 55% B, 5 min increasing from 55 to 75% B, 5 min at 75% B, 5 min decreasing from 75–10% B, and 5 min at 10% B. Venom peak areas were quantified using Shimadzu Lab Solution v2 software, combining default peak calls by the iPeakFinder algorithm with manual curation of the peak baseline and filtering of peaks making up less than 0.1% total area as noise. Peaks were called uniquely per individual snake, by aligning the serial HPLC profiles from all samples from a single individual and calling peaks that were present. The relative abundance of HPLC peaks in each sample will be henceforth referred to as venom composition.

### 4.3. Testosterone ELISA

We used a commercially available EIA kit [62] to measure serum testosterone. Because we anticipated variation between adult and neonate testosterone would differ more than between species, we validated the kit for us in adult and neonate rattlesnakes by pooling serum of both species for adults and neonates separately. Both adult and neonate pools were serially diluted and assessed for parallelism against results from a serial dilution of testosterone at known concentrations to verify the kit’s accuracy across a range of concentrations for adult and neonate serum. We determined the optimal dilution for assessing both age classes simultaneously to be 9:100 unextracted serum to assay buffer. Samples were extracted according to the protocol provided by the kit manufacturer [62]. Briefly, 12 μL serum was extracted by vortexing with diethyl ether at a 5:1 ether:sample ratio, frozen in a dry ice and ethanol bath, and the remaining ether solution pipetted into a new tube. This was repeated for each sample for maximum efficiency. The pooled ether-extractions for each sample were dried in a speedvac, stored at −20 overnight, and redissolved in 133 μL of assay buffer. Samples were plated and treated according to the kit manufacturer protocol [62]. The optical density of each well was read at 450 nm by a SpectraMax iD5 multi-mode microplate reader (Molecular Devices); results are reported in ng/mL. Samples were haphazardly assigned to each plate. There was a subset of samples that did not have sufficient serum to run in duplicate (*n* = 9); for these samples, 6.5 μL of serum was extracted as above and redissolved in 71.5 μL of assay buffer and were run singly. All other samples were run in duplicate, and the values of each duplicate averaged for use in analysis. Samples falling below the limit of detection (*n* = 5) were set at the lower limit of detection for analysis (0.001 ng/mL). The mean intra-assay coefficient of variation was 1.96 ± 1.74% and the inter-assay coefficient of variation was 4.7%.

### 4.4. Dietary Shifts

We accessed previously published diet studies for both *C. horridus* [63] and *C. adamanteus* [64] to assess the extent to which these snakes exist as size-structured populations accessing different resources. Furthermore, each study included SVL of the snakes containing diet items, allowing us to assess the extent to which diet composition changes suddenly or more gradually over time. To do this, we binned the SVLs of both species into quartiles based on the shortest and longest snake in the diet datasets, and tabulated counts of prey in the diet within a given size quartile. Both snakes are known to specialize on mammals, and we were able to categorize the mammals presented in the diet studies into general categories that varied by size [63,64]. In order of smallest to largest average prey size, *C. horridus* fed on shrews, mice, voles, chipmunks, rats, squirrels, and rabbits, while *C. adamanteus* fed on only mice, rats, squirrels, and rabbits. We tested for differences in the overall proportion of these prey classes between adjacent size class quartiles using chi-square tests.

### 4.5. Statistical Analyses

All statistical analyses were performed in R v. 3.6.0 [65]. Venom phenotype as measured using HPLC represents compositional data, and thus exists in the simplex [30,66] and requires transformation prior to analysis. The presence of an ontogenetic shift in the whole venom HPLC profiles of each individual snake was tested with permutational MANOVA as implemented in the adonis function of the vegan 2.5 R package [67], with isometric-log-ratio (ilr) transformed venom composition as a multivariate response and day of study (i.e., time) as a continuous independent variable. To determine the mode of the ontogenetic shift, we visualized venom compositional change by calculating differences between each venom sample and the mean composition of the three earliest juvenile samples taken from a given individual. In this way, our compositional distances for each time point were relative to an average of early composition and not relative to a potentially noisy single first sample. Relative abundance values of zero within a sample were replaced by fractional values using the cmultRepl function in the zcompositions R package [68]. Next, the aDist function in zCompositions was used to calculate the Aitchison compositional distance between the focal sample and the juvenile sample average [68]. The Aitchison distance is a Euclidean distance between data that has been previously subjected to centered log–ratio (clr) transformation, and is an appropriate measure of distance between compositional datasets [66]. Gradual modes of ontogenetic shift are expected to be best fit by a linear model or a sigmoid model with a gradual slope. We used the lm function from base R and the nlsLM function from the minpack.lm package [69] to fit linear regressions and sigmoid curves, respectively, to the relationship between mean-scaled Aitchison distance and time for each snake. We then compared the fit of each model using AICc values. If a nonlinear sigmoid curve was a better fit than a linear model, we used the slope at the sigmoid inflection point (the scale parameter) to determine if 90 percent of the compositional change to the asymptote estimate occurred in less than one active season (number of days from April through the end of November: 244 days). If the change occurred in less than one active season, we counted the peak as having a discrete shift mode, whereas non-linear changes that took longer were counted as gradual. Margres et al. [30] defined support for a discrete shift as any case where a sigmoid curve fits the data better than a linear relationship, and thus our current work is differentiated from Margres et al. [30] in recognizing that a change manifesting as a sigmoid relationship may still take a long time from initiation to asymptote. Last, we placed all samples from all snakes in a comparable multivariate space for visualization in the same analysis of phenotype. To do this, we binned peak areas by retention time eleven retention time windows: R1 (10–20 min), R2 (20–30 min), R3 (30–40 min), R4 (40–50 min), R5 (50–60 min), R6 (60–70 min), R7 (70–80 min), R98 (80–90 min), R9 (90–100 min), R10 (100–110 min), and R11 (110–125 min). Next, we clr-transformed the percent area data for these eleven windows across all 148 venom samples used in this study. We then calculated pairwise Aitchison distance between all samples, and projected them into a 2 dimensional multivariate venom space using NMDS implemented in the metaMDS function in the vegan v.2.5 [67] package in R.

Upon detecting significant shifts in the overall venom profile, we followed with modeling the clr transformed abundance of each peak versus time. This peak-by-peak analysis provided a post-hoc test of which peaks varied in abundance significantly over the course of the study and their mode of change, where mode (no shift, linear shift, gradual sigmoid, or discrete sigmoid) was determined as above using linear and non-linear regression. Non-linear fits were again obtained using the nlsLM function from the minpack.lm package [69].

Last, we determined whether testosterone concentrations were closely associated with the change in venom composition as snakes grew via linear regression. Given the correlative nature of these tests, the best evidence for a potential role of testosterone would be a particularly strong linear relationship between the change in testosterone concentration between the focal and initial reads and the change in venom composition as measured by Aitchison distance. Other forms of a relationship would be inconclusive, as testosterone is expected to be higher in adult than juvenile snakes.

## Figures and Tables

**Table 1:** PERMANOVA results for tests of ontogenetic shifts between each species, with time as a predictor of venom composition.

Snake ID	Species	Df	Sums of Sqs	Mean Sqs	F	R2	*p*-Value
KW1425	*C. adamanteus*	1	61.16	61.16	3.81	0.21	2 × 10^−4^
KW1726	*C. adamanteus*	1	263.08	263.08	20.58	0.58	9.9 × 10^−5^
MM0114	*C. adamanteus*	1	17.76	17.76	4.10	0.23	0.0032
KW1576	*C. horridus*	1	395.08	395.08	16.98	0.49	9.9 × 10^−5^
KW1594	*C. horridus*	1	217.70	217.70	23.43	0.63	9.9 × 10^−5^
MM0027	*C. horridus*	1	102.5	102.5	69.6	0.80	9.9 × 10^−5^
KW1753	*C. horridus*	1	210.43	210.43	14.46	0.41	9.9 × 10^−5^
MM0071	*C. horridus*	1	67.77	67.77	15.25	0.45	9.9 × 10^−5^

**Table 2:** Mode and tempo of the ontogenetic shift. Model summaries for both the linear and sigmoid model fits to the relationship between Aitchison distance and time in each snake. Lowest AIC values are in bold. The tempo of the shift is indicated by the predicted number of days to shift at maximum estimated rate of change. Asterisks indicate ∆AIC values >2. The tempo value with a double asterisk is associated with snakes where the simple linear model was a significantly better fit, and the number of days is simply the total number of days over which that individual was sampled.

		Linear			Sigmoid			
Snake ID	Species	AIC	R2	*p*-Value	AIC	R2	*p*-Value	Tempo (Days)
KW1425	*C. adamanteus*	22.7	0.81	<0.0001	19.1 *	0.88	0.0394	612
KW1726	*C. adamanteus*	**22.8**	0.82	<0.0001	24.3	0.84	0.0087	811
MM0114	*C. adamanteus*	**27.7**	0.71	<0.0001	27.1	0.78	0.0468	213
KW1576	*C. horridus*	29.5	0.76	<0.0001	22.5 *	0.86	0.0005	989
KW1594	*C. horridus*	−0.7 *	0.96	<0.0001	1.7	0.96	0.0001	677 **
MM0027	*C. horridus*	17.6	0.88	<0.0001	−16.7 *	0.98	0.0000	242
KW1753	*C. horridus*	51.7	0.53	<0.0001	44.2 *	0.70	0.0209	309
MM0071	*C. horridus*	**41.3**	0.65	<0.0001	40.9	0.71	0.0254	719

**Table 3:** Number of peaks showing different modes of ontogenetic change in each sampled rattlesnake. The percent gradual is the sum of the “Linear-Gradual” and “Sigmoid-Gradual” count divided by the total number of peaks with statistically significant shifts.

Snake ID	Species	No Shift	Linear-Gradual	Sigmoid-Gradual	Sigmoid-Discrete	Proportion Shifting	Percent Gradual
KW1425	*C. adamanteus*	14	13	1	8	22/36, 61%	64%
KW1726	*C. adamanteus*	7	11	5	7	23/30, 77%	70%
MM0114	*C. adamanteus*	1	2	0	17	19/20, 95%	11%
KW1576	*C. horridus*	10	6	8	5	19/29, 66%	74%
KW1594	*C. horridus*	5	5	7	7	19/24, 79%	63%
MM0027	*C. horridus*	2	3	7	6	16/18, 89%	63%
KW1753	*C. horridus*	10	7	5	4	16/26, 62%	75%
MM0071	*C. horridus*	9	10	1	0	11/20, 55%	100%

**Table 4:** Locality, sex, and size data for the *C. adamanteus* (*n* = 3) and *C. horridus* (*n* = 5) used in this study. CB = Captive born. WC = Wild caught.

Snake ID	Species	Sex	SVL (cm)	Locality	Sample Collection Date Range
KW1425	*C. adamanteus*	F	61–114	WC, Lake Co, FL	21 May 2013–16 February 2017
KW1726	*C. adamanteus*	M	40–110.5	CB, Little St. George, FL	7 October 2013–28 March 2017 *
MM0114 **	*C. adamanteus*	F	37–95	WC, Leon Co, FL	19 November 2014–28 March 2017
KW1576	*C. horridus*	M	34–104.5	CB, Baker Co, GA	20 August 2013–5 July 2018 *
KW1594	*C. horridus*	M	42–89.5	CB, Baker Co, GA	21 September 2013–30 July 2015
MM0027 **	*C. horridus*	M	64–113	WC, Flint River, GA	21 May 2013–1 July 2016
KW1753	*C. horridus*	M	34–100	WC, Baker Co, FL	12 September 2013–16 February 2017
MM0071	*C. horridus*	M	41–100.5	WC, Columbia Co, FL	14 May 2014–16 February 2017

* KW1726 last blood sample date 1 November 2016; KW1576 last blood sample date 28 March 2017. ** Blood samples not assessed.

## Supplementary Materials

The following are available online at https://www.mdpi.com/2072-6651/12/10/659/s1, Figure S1: All HPLC chromatograms used to profile the ontogenetic shifts in the venom of *Crotalus adamanteus*—KW1425, Figure S2: All HPLC chromatograms used to profile the ontogenetic shifts in the venom of *Crotalus adamanteus*— KW1726, Figure S3: All HPLC chromatograms used to profile the ontogenetic shifts in the venom of *Crotalus adamanteus*—MM0114, Figure S4: All HPLC chromatograms used to profile the ontogenetic shifts in the venom of *Crotalus horridus*—KW1576 (Type B venom), Figure S5, All HPLC chromatograms used to profile the ontogenetic shifts in the venom of *Crotalus horridus*—KW1594 (Type B venom), Figure S6: All HPLC chromatograms used to profile the ontogenetic shifts in the venom of *Crotalus horridus*—MM0027 (Type B venom), Figure S7: All HPLC chromatograms used to profile the ontogenetic shifts in the venom of *Crotalus horridus*—KW1753 (Type A venom), Figure S8: All HPLC chromatograms used to profile the ontogenetic shifts in the venom of *Crotalus horridus*—MM0071 (Type A venom), Figure S9: Peak-by-peak analysis of relative abundance shifts over time for an individual *Crotalus adamanteus*, Figure S10: Peak-by-peak analysis of relative abundance shifts over time for an individual *Crotalus adamanteus*, Figure S11: Peak-by-peak analysis of relative abundance shifts over time for an individual type B *Crotalus horridus,* Figure S12: Peak-by-peak analysis of relative abundance shifts over time for an individual type B *Crotalus horridus,* Figure S13: Peak-by-peak analysis of relative abundance shifts over time for an individual type A *Crotalus horridus*, Table S1: Comparison of time (day) and body size (SVL) as predictors of Aitchison distance of venom samples from the juvenile average venom composition. Bold AIC scores indicate the top model for that individual, Table S2: Protein contents of peaks based on HPLC profiles and mass-spectrometry results presented in Wray et al. [10], Table S3: Protein contents of peaks based on HPLC profiles and mass-spectrometry results presented in Wray et al. [10].
