# Supplementary material for: Gradual and Discrete Ontogenetic Shifts in Rattlesnake Venom Composition and Assessment of Hormonal and Ecological Correlates
Source: Toxins (Basel). 2020 Oct 16;12(10):659. doi: 10.3390/toxins12100659 (PMC7602723; doi:10.3390/toxins12100659)
Supplement: Supplementary file 1 [file toxins-12-00659-s001.zip › toxins-920415 Supplementary for conversion.docx]

Supplementary Materials: Gradual and Discrete Ontogenetic Shifts in Rattlesnake Venom Composition and Assessment of Hormonal and Ecological Correlates

Richard B. Schonour, Emma M. Huff, Matthew L. Holding, Natalie M. Claunch, Schyler A. Ellsworth, Michael P. Hogan, Kenneth Wray, James McGivern, Mark J. Margres, Timothy J. Colston and Darin R. Rokyta

**Table S1.** Comparison of time (day) and body size (SVL) as predictors of Aitchison distance of venom samples from the juvenile average venom composition. Bold AIC scores indicate the top model for that individual.

|  |  | **Time Model** | | | | | **Body Size Model** | | | | |
| --- | --- | --- | --- | --- | --- | --- | --- | --- | --- | --- | --- |
| **Snake ID** | **Species** | **Slope** | **T** | ***P*** | **R2** | **AIC** | **Slope** | **T** | ***P*** | **R2** | **AIC** |
| KW1425 | *C. adamanteus* | 0.005 | 8.05 | < 0.00001 | 0.811 | 54.5 | 0.135 | 8.68 | < 0.00001 | 0.832 | **52.51** |
| KW1726 | *C. adamanteus* | 0.010 | 8.64 | < 0.00001 | 0.833 | **67.97** | 0.138 | 7.69 | < 0.00001 | 0.798 | 71.17 |
| MM0114 | *C. adamanteus* | 0.027 | 5.97 | 0.00005 | 0.733 | 89.09 | 0.312 | 6.18 | 0.00003 | 0.746 | **88.33** |
| KW1576 | *C. horridus* | 0.006 | 8.11 | < 0.00001 | 0.771 | 69.46 | 0.120 | 8.43 | < 0.00001 | 0.816 | **65.49** |
| KW1594 | *C. horridus* | 0.014 | 18.09 | < 0.00001 | 0.959 | **32.40** | 0.169 | 16.60 | < 0.00001 | 0.952 | 35.03 |
| MM0027 | *C. horridus* | 0.006 | 11.5 | < 0.00001 | 0.886 | **47.23** | 0.140 | 10.9 | < 0.00001 | 0.873 | 49.19 |
| KW1753 | *C. horridus* | 0.003 | 5.11 | 0.00005 | 0.554 | 82.53 | 0.064 | 6.08 | < 0.00001 | 0.638 | **77.74** |
| MM0071 | *C. horridus* | 0.002 | 6.21 | 0.00001 | 0.670 | 39.75 | 0.036 | 6.95 | < 0.00001 | 0.718 | **36.45** |

**Table S2.** Protein contents of peaks based on HPLC profiles and mass-spectrometry results presented in Wray et al. [10]. Asterisks indicate peaks that we identified in our samples but were not identified in Wray et al. [10].

| **KW1594 – *C. horridus*** | | |
| --- | --- | --- |
| **Present study** | **Wray et al. [10]** | **Proteins** |
| 1 & 2 | 1 | SVMPII-3a, SVMPII-4a, SVSP-6, VEGF-1, BPP-1a/c, HistoneH2AZ/H2A |
| 3 | 2 | SVMPIII-4b, SVMPIII-4a, SVMPII-2 |
| 4* |  |  |
| 5* |  |  |
| 6 | 3 | SVMPIII-4b, SVMPIII-4a, SVMPII-3a, LAAO-1e, LAAO-1b/d |
| 7 | 4a & 4b | MYO, SVMPII-4a, LAAO-1b/d, LAAO-1e, SVMPII-3a, SVSP-6 |
| 8* |  |  |
| 9* |  |  |
| 10* |  |  |
| 11 | 5 | VEGF-1, NGF-1a |
| 12 | 6 | CRISP, NGF-1a, VEGF-1 |
| 13 | 7 | SVSP-2a, SVSP-2b, SVSP-6, PLA2-1d |
| 14 | 9 | PLA2-1c, PLA2-1e, PLA2-1d |
| 15 | 11a & 11b | SVSP-13c, SVSP-13b, PLA2-1d, SVSP-2b |
| 16 | 12 | SVSP-2a, SVSP-2b, SVSP-6, SVSP-11b, SVSP-11d, SVSP-5, CTL-8a, SVSP-1 |
| 17 | 13 | LAAO-1e, PDE, SVSP-6, SVSP-2a, CTL-2 |
| 18 | 14 | Not Given |
| 19 | 15 | Not Given |
| 20* |  |  |
| 21 | 16 | SVMPIII-5a, SVMPIII-2a, SVMPIII-4b, SVMPIII-4a, LAAO-1e |
| 22* |  |  |
| 23* |  |  |
| 24 | 17 | SVMPIII-4a, SVMPIII-4b, SVMPII-3a |

**Table S3:** Protein contents of peaks based on HPLC profiles and mass-spectrometry results presented in Wray et al. [10]. Asterisks indicate peaks that we identified in our samples but were not identified in Wray et al. [10].

| **KW1726 *– Crotalus adamanteus*** | | |
| --- | --- | --- |
| **Present Study** | **Wray et al. [10]** | **Proteins** |
| 1 | 1a | Unidentified |
| 2 | 2 | MYO-1b |
| 3 | 3 | SVMPIII-2b |
| 4 | 4 | SVMPIII-2b |
| 5 | 5 | SVMPIII-2b |
| 6* |  |  |
| 7* |  |  |
| 8* |  |  |
| 9* |  |  |
| 10* |  |  |
| 11* |  |  |
| 12* |  |  |
| 13* |  |  |
| 14, 15, 16 | 8 & 9 | SVSP-5, CRISP |
| 17 | 10 | PLA2-1a/b |
| 18 & 19 | 11 | SVSP-2, SVSP-5 |
| 20 | 12 | SVSP-4 |
| 21 | 13 | SVSP-7, SVSP-4 |
| 22 | 14 | CTL-10, SVSP-4, CTL-11a/b/c |
| 23 | 15a & 15b | CTL-10, CTL-9, SVSP-4, CTL-13f, SVSP-7, LAAO, CTL-11a/b |
| 24 | 16 | LAAO, SVSP-7, SVSP-4, CTL-11a/b/c |
| 25 | 18 | SVMPIII-4d/e |
| 26 | 19 | SVMPIII-3, CTL-11a |
| 27 | 20a | SVMPIII-2b, SVMPII-2c/d, SVMPII-2b |
| 28 | 20b | SVMPII-2b, LAAO, CTL-10, CTL-11a/b, SVMPIII-2b, CTL-13f, SVSP-4 |
| 29* |  |  |
| 30 | 21 | SVMPII-1a/b/c/d |

Toxin protein abbreviations: BPP = bradykinin-potentiating peptide, CTL = C-type lectins, CRISP = cysteine-rich secretory protein, LAAO = L-amino oxid, MYO = myotoxin-A, NGF = nerve growth factor, PDE = phosphodiesterase, PLA2 = phospholipase A2, SVMP = snake venom metalloproteinase (types II and III), SVSP = snake venom serine proteinase, VESP = vespryn, VEGF = vascular endothelial growth factor.

**Figure S1.** All HPLC chromatograms used to profile the ontogenetic shifts in the venom of *Crotalus adamanteus* – KW1425. Day corresponds with day of the study starting with day 0 as the time of first juvenile venom extraction. Numbers starting with V correspond to unique venom sample numbers. Numbers on the peaks in the top, left chromatogram correspond to the numbers assigned to called peaks, and are the same as in Figure 1 of the main text.

**Figure S2.** All HPLC chromatograms used to profile the ontogenetic shifts in the venom of *Crotalus adamanteus* – KW1726. Day corresponds with day of the study starting with day 0 as the time of first juvenile venom extraction. Numbers starting with V correspond to unique venom sample numbers. Numbers on the peaks in the top, left chromatogram correspond to the numbers assigned to called peaks, and are the same as in Figure 1 of the main text.

**Figure S3.** All HPLC chromatograms used to profile the ontogenetic shifts in the venom of *Crotalus adamanteus* – MM0014. Day corresponds with day of the study starting with day 0 as the time of first juvenile venom extraction. Numbers starting with V correspond to unique venom sample numbers. Numbers on the peaks in the top, left chromatogram correspond to the numbers assigned to called peaks, and are the same as in Figure 1 of the main text.

**Figure S4.** All HPLC chromatograms used to profile the ontogenetic shifts in the venom of *Crotalus horridus* – KW1576 (Type B venom). Day corresponds with day of the study starting with day 0 as the time of first juvenile venom extraction. Numbers starting with V correspond to unique venom sample numbers. Numbers on the peaks in the top, left chromatogram correspond to the numbers assigned to called peaks, and are the same as in Figure 1 of the main text.

**Figure S5.** All HPLC chromatograms used to profile the ontogenetic shifts in the venom of *Crotalus horridus* – KW1594 (Type B venom). Day corresponds with day of the study starting with day 0 as the time of first juvenile venom extraction. Numbers starting with V correspond to unique venom sample numbers. Numbers on the peaks in the top, left chromatogram correspond to the numbers assigned to called peaks, and are the same as in Figure 1 of the main text.

**Figure S6.** All HPLC chromatograms used to profile the ontogenetic shifts in the venom of *Crotalus horridus* – MM0027 (Type B venom). Day corresponds with day of the study starting with day 0 as the time of first juvenile venom extraction. Numbers starting with V correspond to unique venom sample numbers. Numbers on the peaks in the top, left chromatogram correspond to the numbers assigned to called peaks, and are the same as in Figure 1 of the main text. Note: the large noise at the beginning of profile V0885-Day 246 (minutes 0-10) shifted the this entire profile down in total height, but was not counted during peak relative abundance determination.

**Figure S7.** All HPLC chromatograms used to profile the ontogenetic shifts in the venom of *Crotalus horridus* – KW1753 (Type A venom). Day corresponds with day of the study starting with day 0 as the time of first juvenile venom extraction. Numbers starting with V correspond to unique venom sample numbers. Numbers on the peaks in the top, left chromatogram correspond to the numbers assigned to called peaks, and are the same as in Figure 1 of the main text. Note: the large noise at the beginning of profile V0805-Day 81 and V0819 (minute 3) shifted the this entire profile down in total height, but was not counted during peak relative abundance determination.

**Figure S8.** All HPLC chromatograms used to profile the ontogenetic shifts in the venom of *Crotalus horridus* – MM0071 (Type A venom). Day corresponds with day of the study starting with day 0 as the time of first juvenile venom extraction. Numbers starting with V correspond to unique venom sample numbers. Numbers on the peaks in the top, left chromatogram correspond to the numbers assigned to called peaks, and are the same as in Figure 1 of the main text.

**Figure S9.** Peak by peak analysis of relative abundance shifts over time for an individual *Crotalus adamanteus*. Panels with best-fit lines indicate peaks that changed significantly in abundance over time. Black lines indicate best-fit of a linear model to the data, while blue lines show cases where a non-linear fit was best.

**Figure S10.** Peak by peak analysis of relative abundance shifts over time for an individual *Crotalus adamanteus*. Panels with best-fit lines indicate peaks that changed significantly in abundance over time. Black lines indicate best-fit of a linear model to the data, while blue lines show cases where a non-linear fit was best.

**Figure S11.** Peak by peak analysis of relative abundance shifts over time for an individual type B *Crotalus horridus*. Panels with best-fit lines indicate peaks that changed significantly in abundance over time. Black lines indicate best-fit of a linear model to the data, while blue lines show cases where a non-linear fit was best.

**Figure S12.** Peak by peak analysis of relative abundance shifts over time for an individual type B *Crotalus horridus*. Panels with best-fit lines indicate peaks that changed significantly in abundance over time. Black lines indicate best-fit of a linear model to the data, while blue lines show cases where a non-linear fit was best.

**Figure S13.** Peak by peak analysis of relative abundance shifts over time for an individual type A *Crotalus horridus*. Panels with best-fit lines indicate peaks that changed significantly in abundance over time. Black lines indicate best-fit of a linear model to the data, while blue lines show cases where a non-linear fit was best.
